# A simplified vocal tract model for articulation of [s]: The effect of tongue tip elevation on [s]

**DOI:** 10.1371/journal.pone.0223382

**Published:** 2019-10-10

**Authors:** Tsukasa Yoshinaga, Kazunori Nozaki, Shigeo Wada

**Affiliations:** 1 Toyohashi University of Technology, Toyohashi, Aichi, Japan; 2 Osaka University Dental Hospital, Suita, Osaka, Japan; 3 Graduate School of Engineering Science, Osaka University, Toyonaka, Osaka, Japan; University Hospital Eriangen at Friedrich-Alexander-University Erlangen-Numberg, GERMANY

## Abstract

Fricative consonants are known to be pronounced by controlling turbulent flow inside a vocal tract. In this study, a simplified vocal tract model was proposed to investigate the characteristics of flow and sound during production of the fricative [s] in a word context. By controlling the inlet flow rate and tongue speed, the acoustic characteristics of [s] were reproduced by the model. The measurements with a microphone and a hot-wire anemometer showed that the flow velocity at the teeth gap and far-field sound pressure started oscillating before the tongue reached the /s/ position, and continued during tongue descent. This behaviour was not affected by the changes of the tongue speed. These results indicate that there is a time shift between source generation and tongue movement. This time shift can be a physical constraint in the articulation of words which include /s/. With the proposed model, we could investigate the effects of tongue speed on the flow and sound generation in a parametric way. The proposed methodology is applicable for other phonemes to further explore the aeroacoustics of phonation.

## Introduction

**Fig 1:**
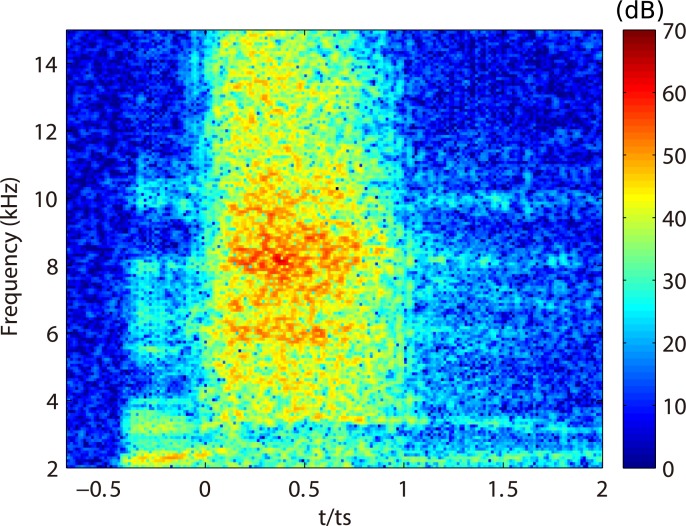
Example of spectrogram of fricative /s/ in a word /usui/. The word was pronounced by a male Japanese subject. The sound of [s] appears from *t*/*t*_s_ = 0 to 1 (*t*_s_ = 175 ms). The original audio file is in the supplemental material (S1 Audio).

Fricative consonants are known to be produced by using turbulent jet flow and its aeroacoustic sound source in a vocal tract [1]. The jet flow is generated at the constricted flow channel formed by the anterior portion of the tongue and hard palate. When fricatives are generated in the production of words, the aeroacoustic sound appears as a broad-band noise above 4 kHz [2] within 100–200 ms before and/or after the vowel is produced [3]. An example of the spectrogram of fricative /s/ in a word /usui/ (“*thin”* in Japanese) is shown in Fig 1. The original audio file is in the supplemental material (S1 Audio).

The articulatory mechanisms involved in producing a [s] sound have been investigated by measuring the tongue movement using electro-magnetic sensors [3–4], medical images [5–6], and measuring the glottal opening using an optical sensor [7]. Measurement based on electro-palatography [3] indicates that tongue contact on the hard palate occurs after the appearance of fricative noise, whereas the duration of the sound is longer than the duration of the tongue contact. Although the measurement revealed a time shift of the sound generation to the tongue movement, the mechanisms and timing of the aeroacoustic sound generation are still unclear.

The sound source of [s] is mainly generated by the impingement of the jet on an obstacle downstream from the constriction, *i*.*e*., front teeth and lips [8]. Thus, it is believed that dipole sources appear on the obstacle walls in the vocal tract, and the sound source was modeled by assuming simple flow configuration downstream from the constriction in theoretical work. Krane [9] proposed a theory of vortex sound in the vocal tract using configuration of vortex rings of the jet flow and a simplified duct with an obstacle. In addition, the acoustic characteristics of [s] were investigated by assuming the sound source near the front teeth wall in a simplified vocal tract of [s] [10–11]. Meanwhile, numerical flow simulations were applied to several vocal tract geometries [12–14], and results showed that the sound source of velocity fluctuation is widely distributed from the upper teeth wall to the lower lip surface.

However, almost all simulation studies have considered only the steady flow inlet and channel, and few studies have been reported on the effects of tongue movement on the appearance and disappearance of the sound source, including laminar-turbulent transition control inside the vocal tract. Since both the inlet flow rate and the flow channel of the vocal tract are dynamically changed while pronouncing [s] in the word context, it is necessary to examine the tongue movement, flow configuration inside the vocal tract, and far-field sound characteristics simultaneously to clarify the production mechanisms. To do so, we conducted experimental measurements on the flow and sound of [s] in the word context /usui/ using a simplified vocal tract model, in which the inlet flow rate and tongue movement can be controlled.

## Materials and methods

**Fig 2:**
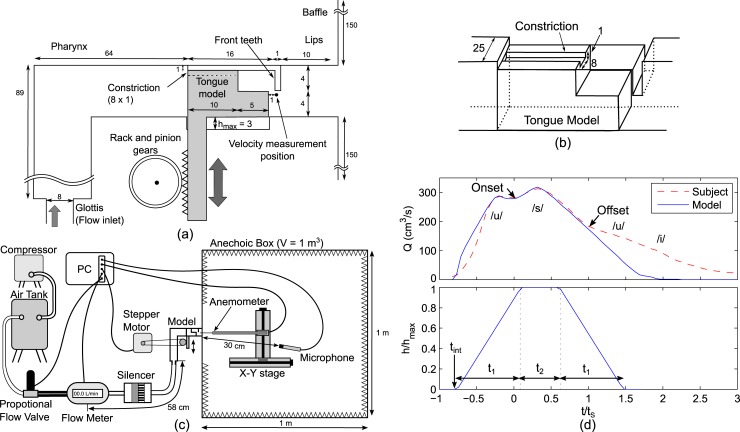
Experimental setups. (a) Simplified vocal tract model of [s] (unit of dimensions mm). (b) Three-dimensional schematic of the tongue model of the simplified model. (c) Schematics of the experimental setups. (d) Flow rate *Q* and tongue height *h*/*h*_max_ for the experiment. The flow rate of the model was compared with that of the subject pronouncing /usui/.

The simplified vocal tract model was constructed based on magnetic resonance images of a Japanese male producing [s] [15]. The simplified vocal tract model [Fig 2 (A)] consists of a rectangular flow channel representing five cross-sectional shapes (pharynx, constriction, alveolar ridge, teeth gap, and lips, which are significant in producing [s] [16]) in the vocal tract of the subject. The flow channel at the pharynx was bent in a perpendicular direction to the front part of the vocal tract. On the upper surface of the tongue model, a constricted flow channel (8 × 1 mm) was formed at the center in transverse direction in the model. The three-dimensional schematic of tongue model is shown in Fig 2(B). Although the tongue constriction has an unrealistic rectangular inlet and outlet, we confirmed that the flow configuration of the jet flow downstream from the constriction is similar to that of the realistic geometry of [s] by performing the numerical flow simulation [14].

The tongue model was connected to the rack gear and moved up and down by rotating a pinion gear from the position of /u/ (*h* = 0) to the position of /s/ (maximum height: *h*_max_ = 3 mm). Downstream from the lip cavity, a baffle was set to imitate a subject's face. This model can reproduce the sound of [s] in the frequency range from 2 to 15 kHz when the tongue model is at the /s/ position (*h*/*h*_max_ = 1) [15].

Referring to the experimental setups [Fig 2(C)], which include the model and measurement equipment, the rotational movement of the pinion gear is controlled by a stepper motor (two-phase, 0.9^o^, ST-42BYH1004, Mercury Motor, Japan) connected with a timing belt. The air was inserted from a compressor (YC-4RS, Yaezaki, Japan) to the model through an air tank, a proportional electro-magnetic flow valve (PVQ30, SMC, Japan), a mass flow meter (Series 4043, TSI, Rochester, NY, frequency range up to 100 Hz), and a silencer (volume: 500 cm^3^) connected with air tubes of inner diameter 16 mm. The simplified model has a smaller inner diameter 8 mm at the inlet. The electro-magnetic valve changes the volume flow rate *Q* proportionately to the input voltage. To reproduce the flow rate of /s/ during pronunciation of /usui/, we used the mass flow meter (same type as described above) and a venturi mask (Acurox type, Japan Medicalnext Co., Ltd., Japan) to measure the flow rate produced by the subject. Then, the recorded flow rate was reproduced by changing the voltage of the valve. The flow rates of the subject and model, as well as the track of the tongue height, are plotted in Fig 2(D). Time *t* was normalized by the mean duration of [s] (*t*_s_ = 175 ms). The onset and offset of [s] were determined based on the time when the amplitude of the sound at 9 kHz in spectrogram exceeds 15 dB. The subject's flow rate is an average of twelve repeated measurements. The flow rate was first increased from 280 cm^3^/s at the end of /u/ (*t*/*t*_s_ = 0) to the maximum of 313 cm^3^/s at *t*/*t*_s_ = 0.314, then decreased to 184 cm^3^/s at the end of [s] (*t*/*t*_s_ = 1). Since the response of the flow rate changes with dynamic control of the vocal tract, we adjusted the input voltage until the flow rate of the model matched with that of the subject. A silencer was set upstream from the model to suppress noise from the air valve. The onset of *Q* was matched with the onset of velocity of the hot-wire measurement to remove delay of flow propagation through the air tubes.

The tongue ascent and descent were modeled with the linear movement for three tongue speeds. The initial timing *t*_int_, tongue moving period *t*_1_, and duration for the maximum tongue height *t*_2_ [Fig 2(D)], for the three tongue speeds are listed in Table 1. In a preliminary measurement with MR movie, the subject was pronouncing /usui/ with tongue speed approximately 30 mm/s (see supplemental material, S1 Video). Therefore, tongue speed was varied from 10 to 40 mm/s to assess the effects on the flow and sound generation. The timing parameters were chosen by conducting preliminary experiments repeatedly until the model produces the sound for *t*_s_ = 175 ms. First, the flow rate *Q* and far-field sound were measured simultaneously, and *t*_int_ was determined to match the onset of the sound generation with the onset of [s] in the flow rate (the trough of *Q* in Fig 2(D)). Then, since *t*_1_ can be calculated with *h*_max_ divided by the tongue speed, *t*_2_ was adjusted to make the sound duration similar to that of the subject *t*_s_ = 175 ms.

**Table 1:** Initial timing and duration of tongue movement for three tongue speeds.

Tongue Speed (mm/s)	*t*_1_/*t*_s_	*t*_2_/*t*_s_	*t*_int_/*t*_s_
40	0.429	0.714	−0.383
20	0.857	0.514	−0.760
10	1.714	0.200	−1.457

The flow velocity downstream from the teeth gap and far-field sound pressure were measured by an anemometer (0251R-T5, Kanomax, Japan) and a microphone (Type 4939, Bruel & Kjaer, Denmark), respectively. The outlet of the simplified model was positioned inside an anechoic box, and the sound was measured at 30cm from the outlet using the microphone. From numerical simulations on this simplified vocal tract, we found that the maximum amplitude of the sound source appeared downstream from the teeth gap [14]. Therefore, a tip of the anemometer was positioned 1 mm below the upper front teeth and 1 mm downstream from the tongue model [Fig 2(A)], to capture the emergence of the sound source. The position of the anemometer was adjusted using X-Y stages (LS-4042-S1; ALS-115-E1P, Chuo Precision Industrial, Japan). The anemometer was calibrated in a small wind tunnel (Model 1065, Kanomax, Japan) every 1 m/s from 2 to 10 m/s, and every 5 m/s from 10 to 50 m/s using a power law [17].

The sound and velocity were recorded with sampling frequency 44 kHz using a data acquisition system (PXIe-4492, National Instruments, Austin, TX). The flow rate *Q* at the inlet of the model was recorded with sampling frequency 100 Hz at the same time. The stepper motor and flow valve were controlled using a micro-controller (Arduino Uno) connected to a computer. Spectrograms of the recorded sound were calculated using a fast Fourier transform with 30% overlapped 512-point signal windows multiplied by a Hanning window. The sound pressure level (SPL) is based on the reference level 20 × 10^−6^ Pa. To evaluate the amplitude of the sound produced as [s], the overall SPL (OASPL) was calculated in the frequency range above the subject’s first characteristic peak from 4 to 15 kHz. To see effects of the inlet flow control and the tongue ascent speed on the sound generation, we calculated the velocity at the constriction,

$$\begin{array}{c} \bar{U}=Q/A_{c}, \end{array}$$ (1)

where *A*_c_ is the area of the flow channel at the constriction (= 8 + 25 × (3—*h*) mm^2^).

## Results

**Fig 3:**
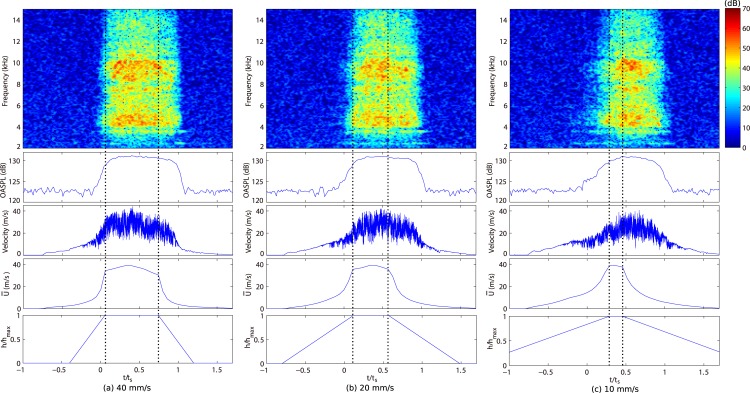
Time variation of sound and flow in the simplified model. Spectrogram (top), OASPL (upper middle), flow velocity (lower middle), and velocity at the constriction $\bar{U}$ (bottom) are shown for three tongue speed (a) 40 mm/s, (b) 20 mm/s, and (c) 10 mm/s. The tongue height is plotted in the bottom and the interval of complete constriction (*t*_2_/*t*_s_) is plotted with vertical dashed lines. The audio, velocity, and flow data are in the supplemental materials S1 Audio, S4 and S2 Datasets for 40 mm/s, S3 Audio, S3 and S4 Datasets for 20 mm/s, and S4 Audio, S5 and S6 Datasets for 10 mm/s.

Results of sound and velocity measurements for three tongue speeds are summarized in Fig 3. In the spectrograms of the generated sound (Fig 3, top), large amplitudes above 4 kHz appeared when 0 ≤ *t/t*_s_ ≤ 1.0. With a tongue speed of 20 mm/s, amplitudes in the frequency range from 4 to 15 kHz increased from *t/t*_s_ = 0, reaching a maximum at *t/t*_s_ = 0.49, and then decreased until *t/t*_s_ = 1.0. The spectrogram for each tongue speed shows that the duration of the maximum amplitude is shortened as tongue speed decreases. This arises with the different *t*_2_ for each tongue speed. The reason is that *t*_2_ gets shorter for lower tongue speeds. The tongue height reached a maximum when 0.05 ≤ *t/t*_s_ ≤ 0.76 for 40 mm/s, 0.10 ≤ *t/t*_s_ ≤ 0.61 for 20 mm/s, and 0.26 ≤ *t/t*_s_ ≤ 0.46 for 10 mm/s. Compared with the amplitudes with 40 mm/s, the amplitudes in the lower frequency range (around 4 to 10 kHz) increased earlier than those in the higher frequency range (10 to 15 kHz) when the tongue speed is 10 mm/s.

The time variation of OASPLs (Fig 3, upper middle) showed that the OASPL over 60 dB lasted for 0.12 ≤ *t/t*_s_ ≤ 0.92 for 40 mm/s, 0.16 ≤ *t/t*_s_ ≤ 0.87 for 20 mm/s, and 0.30 ≤ *t/t*_s_ ≤ 0.84 for 10 mm/s. The flow velocity measured downstream from the teeth gap (Fig 3, lower middle) exhibited fluctuations of large magnitude appearing while the sound is generated. In contrast, small velocity fluctuations proceeded the sound generation. The velocity fluctuation started when *t/t*_s_ = −0.23 for 40 mm/s, *t/t*_s_ = −0.27 for 20 mm/s, and *t/t*_s_ = −0.30 for 10 mm/s. The velocity at the constriction (Fig 3, bottom) first increased rapidly up to around $\bar{U}$ = 34 m/s while the tongue was ascended and then increased gradually with increments of *Q* as the tongue reached maximum height. After reaching a maximum of $\bar{U}$ = 39 m/s, $\bar{U}$ first gradually decreased with decreasing *Q* and then rapidly decreased with tongue descent. The earlier emergence of the velocity fluctuation and sound generation in the lower frequencies with a tongue speed of 10 mm/s correlated with higher $\bar{U}$ in *t/t*_s_ < 0, which had increased with the higher tongue position of 10 mm/s compared with tongue speeds 20 and 40 mm/s. All measured data are in the supplemental materials S1 Audio, S1 and S2 Datasets for 40 mm/s, S3 Audio, S3 and S4 Datasets for 20 mm/s, and S4 Audio, S5 and S6 Datasets for 10 mm/s.

**Fig 4:**
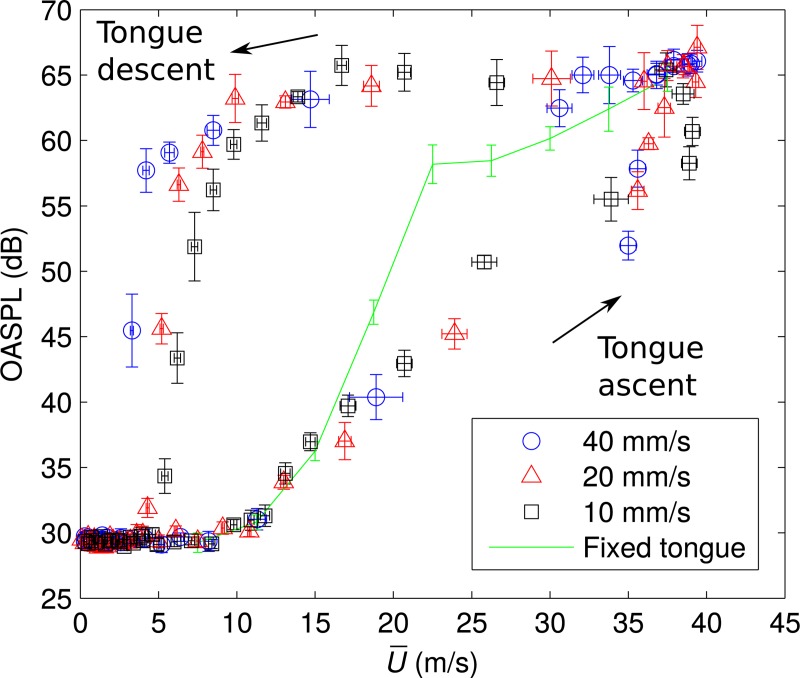
OASPLs of generated sound at each corresponding velocity $\bar{U}$ for three tongue speeds. The bars are standard deviation of five repetitive measurements. OASPLs with the fixed tongue position (maximum tongue height *h/h*_max_ = 1) are also plotted (solid line).

To clarify the relationship of timing between the sound generation and the flow control with tongue movement, OASPLs at each $\bar{U}$ are plotted in Fig 4. The bars are standard deviation of five repetitive measurements. During tongue ascent, the amplitude of the sound started increasing at $\bar{U}$ = 10 m/s. When the tongue model reached *h/h*_max_ = 1, $\bar{U}$ was around 35 m/s with tongue speeds 20 and 40 mm/s. Then, the OASPL increased from 55 to 65 dB while incrementing $\bar{U}$ up to 39 m/s. In contrast, while *h/h*_max_ = 1 with a tongue speed of 10 mm/s, $\bar{U}$ decreased from 39 to 37 m/s while incrementing OASPL from 58 to 65 dB. During tongue descent, $\bar{U}$ decreased rapidly from 39 to 10 m/s with OASPL decreasing from 65 to 60 dB for the three tongue speeds. Then, the OASPL decreased rapidly from 60 to 30 dB when $\bar{U}$ was around 5 m/s.

OASPLs of the sound generated by the model when the tongue model was fixed at a maximum height *h/h*_max_ = 1 are also plotted in Fig 4. The $\bar{U}$ was varied by changing *Q* for the fixed tongue position; note though that *Q* was fixed during recordings. During tongue ascent, amplitudes were consistent when $\bar{U}$ ≤ 15 m/s and became smaller than those of the fixed tongue position when $\bar{U}$ > 15 m/s. In contrast, during tongue descent, amplitudes were larger than those for a fixed tongue position.

## Discussion

**Fig 5:**
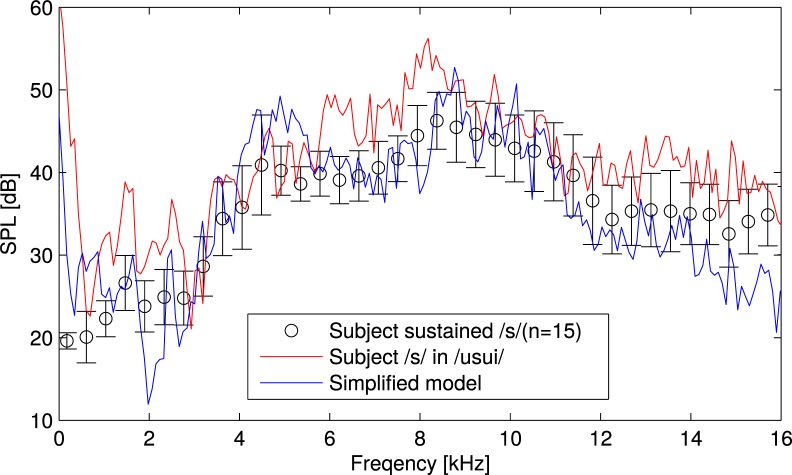
Spectra of /s/ extracted from the spectrogram of /usui/ and the sound generated by the simplified model. The subject’s sustained [s] is also plotted with error bars of 15 repetitive measurements.

The acoustic characteristics in the spectrograms were consistent with those observed in the spectrogram of /s/ in context /usui/ (Fig 1) and also the other word context [7]. This result indicates that the proposed simplified model is capable of producing the fricative /s/ in word contexts. Meanwhile, the spectral peaks at 6 kHz and 8 kHz observed in the spectrogram of /s/ in the word /usui/ were different in the peaks 4 kHz and 10 kHz of the simplified model. To clarify the differences of the peaks between the simplified model and subject’s sound, spectra extracted from the spectrograms are plotted in Fig 5. As described in the method, the model was firstly designed to reproduce the sustained [s]. The subject’s sustained [s] is also plotted with error bars of 15 repetitive measurements. As seen in the spectra, the simplified model reproduced the subject’s frequency peak at 4 kHz and 10 kHz. In contrast, when the subject pronounced /s/ in the word context /usui/, the peaks appeared at 6 kHz and 8 kHz. This indicates that the vocal tract geometry of [s] was changed by the vowel context of /u/. To further investigate the differences of vocal tract geometry, we need to measure the vocal tract geometry of /s/ in the word context /usui/.

**Fig 6:**
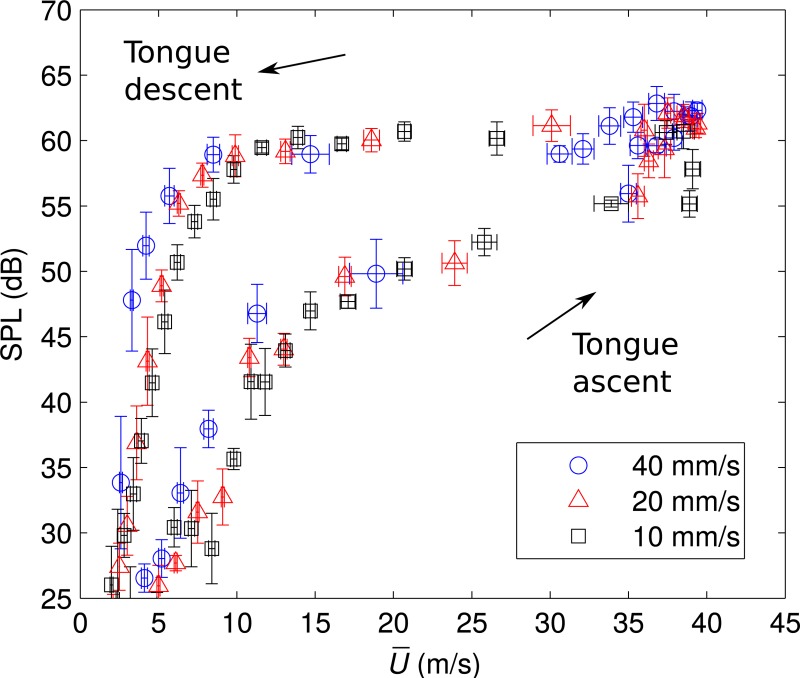
Estimated pressure amplitude (Eq (2)) at each corresponding velocity $\bar{U}$ for three tongue speeds. The bars are standard deviation of five repetitive measurements for $\bar{U}$ and *U*_*c*_.

To see the relationship between the flow velocity and sound amplitude in the model, acoustic pressure was estimated based on the theory proposed by Krane [9]. The pressure amplitude was calculated by vortex rings passing through the axisymmetric cylinder:

$$p^{\prime }∼\rho_{\infty }\frac{R_{v}\delta_{v}}{R_{min}^{2}}U_{c}^{2}\frac{R_{p}-R_{min}}{({\left(R_{p}-R_{min}{)}^{2}+H^{2}\right)}^{1/2}},$$ (2)

where, *p*′ is acoustic pressure, *ρ*_∞_ is air density, *R*_*v*_, *δ*_*v*_, and *U*_*c*_ are radius, core diameter, and convection speed of the vortex ring, *R*_min_ and *R*_*p*_ are radius of constriction and duct away from the constriction, *H* is axial length of the constriction. Although the theory is limited for simple axisymmetric cylinder and for low frequency sound, this equation was used to clarify the relationship between the measured velocity and the sound. From the geometry of the simplified model in this paper, the dimensions were determined as *R*_min_ = (4 –*h*)/2 mm, *R*_*p*_ = 4 mm (half length of the lip height), and *H* = 1 mm (gap between teeth). The size of vortex ring was estimated as *R*_*v*_ = 0.1 mm and *δ*_*v*_ = 0.05 mm based on vortex tubes shown in the flow simulation [14]. The velocity *U*_*c*_ was estimated by calculating root mean square values of measured velocity (hot-wire) with 512-point signal windows of the spectrogram. The estimated pressure amplitudes are plotted with velocity at the constriction in Fig 6.

The overall behavior of SPLs estimated by Eq (2) agreed well with the measured OASPLs (Fig 4) for both tongue ascent and descent. According to Eq (2), the amplitude of generated sound depends on the sizes of the vortex ring and constriction, and magnitude of the convection velocity. In this study, the changes in OASPLs during tongue motion were well predicted by considering only the changes of jet velocity *U*_*c*_ and the length of maximum constriction *R*_min_. Since the change of *R*_min_ is much smaller than those of *U*_*c*_, the theory suggests that the amplitude mainly depends on the magnitude of *U*_*c*_ formed by the tongue motion and flow rate *Q*.

During tongue elevation, although the sound of [s] appeared before the tongue reached the maximum height *h*/*h*_max_ = 1, the amplitudes were smaller than those measured during tongue descent and fixed tongue position at same $\bar{U}$. These differences were caused by smaller magnitudes of *U*_*c*_ during tongue elevation (*e*.*g*., *U*_*c*_ = 3.02 m/s during tongue elevation *t*/*t*_s_ = 0.17, whereas *U*_*c*_ = 4.54 m/s during tongue descent *t*/*t*_s_ = 0.51 at $\bar{U}$ = 26 m/s with tongue speed 10 mm/s). This indicates that there is a delay to form the large convection velocity at the teeth region from the tongue elevation and the increase of $\bar{U}$ at the constriction.

In contrast, OASPLs during tongue descent were larger than those of fixed tongue position. During tongue descent, $\bar{U}$ rapidly decreases since the flow rate *Q* decreases and the cross-sectional area *A*_c_ increases at the same time. However, with increasing *A*_c_, flow pressure drops rapidly at the tongue constriction and the flow velocity near the teeth remains large and continuously fluctuating. Therefore, the velocities *U*_*c*_ remained large and the sound was generated continuously during tongue descent. These explain the longer duration of the sound generation compared with that of tongue contact [3].

## Conclusions

In this study, a simplified vocal tract model was proposed to investigate the relationship between tongue movement and sound generation in the articulation of [s]. The proposed model reproduced the acoustic characteristics of [s] in the word context. With the hot-wire measurements, we found that the sound generation preceded the tongue contact because the flow velocity and its sound source started fluctuating near the teeth gap during tongue ascent. Meanwhile, the sound was generated continuously during tongue descent because the flow velocity remained large at the teeth gap by the pressure drop at the tongue constriction. These results were consistent with three tongue speeds. This indicates that we unconsciously control the tongue movement and flow state inside the vocal tract to generate the turbulent source for the appropriate duration in word pronunciation. Moreover, these results suggest that the observed flow conditions can be a physical constraint for the variation in articulation of the following vowel or consonant [5]. In the previous studies of phonetics with human subjects, the tongue was moved with subject’s intention, and tongue speed could not be controlled. In contrast, by using the proposed model, we could investigate effects of the tongue speed on the flow and sound generation in a parametric way. For further study, the proposed model and methodology are applicable to other phonemes to clarify the aeroacoustics of phonation. In addition, to further analyse the relationship between the generated sound and flow, it is necessary to clarify the configuration of flow and sound source during tongue movement by conducting numerical simulations [14] using the proposed model.

## Supporting information

S1 Audio Sound of /usui/ pronounced by the subject. The sound was recorded with sampling frequency 44100 Hz.
(WAV)

S2 Audio Sound of [s] produced by the model with tongue speed 40 mm/s. (WAV)

S3 Audio Sound of [s] produced by the model with tongue speed 20 mm/s. (WAV)

S4 Audio Sound of [s] produced by the model with tongue speed 10 mm/s. (WAV)

S1 Video MR movie of the subject pronouncing /usui/. The images were scanned with frame rate 13 fps.
(AVI)

S1 Dataset Velocity measured by hot-wire anemometry with tongue speed 40 mm/s. (TXT)

S2 Dataset Inlet flow rate measured by mass flow meter with tongue speed 40 mm/s. (TXT)

S3 Dataset Velocity measured by hot-wire anemometry with tongue speed 20 mm/s. (TXT)

S4 Dataset Inlet flow rate measured by mass flow meter with tongue speed 20 mm/s. (TXT)

S5 Dataset Velocity measured by hot-wire anemometry with tongue speed 10 mm/s. (TXT)

S6 Dataset Inlet flow rate measured by mass flow meter with tongue speed 10 mm/s. (TXT)
